# Ablation of caspase-1 protects against TBI-induced pyroptosis in vitro and in vivo

**DOI:** 10.1186/s12974-018-1083-y

**Published:** 2018-02-19

**Authors:** Wei Liu, Yuhua Chen, Jiao Meng, Minfei Wu, Fangfang Bi, Cuicui Chang, Hua Li, Liangjun Zhang

**Affiliations:** 1 Department of Medical Science Research Center, Peihua University, Xi’an, 710125 People’s Republic of China; 2 Department of Medical Science Research Center, Shaanxi Fourth People Hospital, Xi’an, 710043 People’s Republic of China; 3 Department of Orthopedics, Jilin University Second Hospital, Changchun, 8974617 People’s Republic of China

**Keywords:** TBI, Inflammation, Neuroinflammation, Pyroptosis, Caspase-1, Neuron damage

## Abstract

**Background:**

Traumatic brain injury (TBI) is a critical public health and socioeconomic problem throughout the world. Inflammation-induced secondary injury is one of the vital pathogenic parameters of TBI. Molecular signaling cascades of pyroptosis, a specific type of cellular necrosis, are key drivers of TBI-induced inflammation.

**Methods:**

In this study, mice with genetically ablated caspase-1 (caspase-1^−/−^) were subjected to controlled cortical impact injury in vivo, and primary neuron deficient in caspase-1 through siRNA knockdown and pharmacologic inhibition was stimulated by mechanical scratch, equiaxial stretch, and LPS/ATP in vitro. We evaluated the effects of caspase-1 deficiency on neurological deficits, inflammatory factors, histopathology, cell apoptosis, and pyroptosis.

**Results:**

During the acute post-injury period (0–48 h) in vivo, motor deficits, anti-inflammatory cytokines (TGF-β and IL-10), pro-inflammatory cytokines (IFN-γ, IL-1β, and IL-18), and blood lactate dehydrogenase (LDH), as well as pyroptosis-related proteins (caspase-1, caspase-1 fragments, caspase-11 and GSDMD), were increased. Caspase-1 was activated in the cortex of TBI mice. Inflammatory activation was more profound in injured wild-type mice than in caspase-1^−/−^ mice. In vitro, mechanical scratch, equiaxial stretch, and LPS/ATP-induced neuron pyroptosis, apoptosis, LDH release, and increased expression of inflammatory factors. The effects of mechanical and inflammatory stress were reduced through inhibition of caspase-1 activity through siRNA knockdown and pharmacologic inhibition.

**Conclusion:**

Collectively, these data demonstrate that pyroptosis is involved in neuroinflammation and neuronal injury after TBI, and ablation of caspase-1 inhibits TBI-induced pyroptosis. Our findings suggest that caspase-1 may be a potential target for TBI therapy.

**Electronic supplementary material:**

The online version of this article (10.1186/s12974-018-1083-y) contains supplementary material, which is available to authorized users.

## Background

Traumatic brain injury (TBI) is a type of physical brain insult caused by blunt mechanical force [1]. According to the World Health Organization, TBI is a critical public health and socioeconomic problem throughout the world. Considering the incidence of TBI increases annually, TBI will be a major health problem and a substantial cause of disabilities by 2020 [2, 3]. Treatment of TBI can be very costly due to the need for on-going treatment of long-term effects, which can include psychiatric vulnerability and neurological disorders. The prevalence of neurological dysfunction following TBI has been increasingly appreciated [4, 5]. For example, the risk of clinical depression is approximately1.5 times higher in TBI survivors compared to the general population. Furthermore, the risk for developing Alzheimer disease is 2.3 to 4.5 times greater, depending on whether the TBI is moderate or severe [6]. Worse, still no effective treatment options are available, and little is known about the complex cellular response to TBI.

TBI occurs when the brain is exposed to external forces that induce focal and/or diffuse injuries that include axonal shearing, edema, vascular damage, and neuronal death [7–9]. Injuries from TBI can range from mild to severe [10]. The post-TBI primary insult typically leads to secondary damage and neuronal death caused by inflammation [8, 9, 11], oxygen radical formation [12, 13], calcium release [14], and mitochondrial dysfunction [15, 16]. To date, TBI research outcomes have not translated into clinical therapeutic approaches [17]. Thus, there is an increasing need for further research into the signaling cascades activated by TBI.

Neuroinflammatory signaling leads to a specific type of inflammatory necrosis called pyroptosis, which is characterized by cell swelling, lysis, and release of pro-inflammatory cytokines and intracellular contents [18]. Pyroptosis is involved in pathological processes during viral or bacterial infection (or in the presence of products from pathogens such as lipopolysaccharides (LPS) or viral DNA) [19, 20], myocardial ischemia, lung and kidney injury, and cerebral stroke [21–24]. This type of inflammatory necrosis occurs predominantly in the phagocytes, macrophages, monocytes, and dendritic cells, as well as various other cell types such as T cells. The pro-inflammatory caspase subfamily, including caspase-1 in humans and mice, caspase-4 and caspase-5 in humans, and caspase-11 in mice, mediate pyroptosis [19]. Li et al. found that chemical neurotoxicity-induced pyroptosis of the cortical neurons is dependent on caspase signaling through the Erk1/2-Nrf2/Bach1 signal pathway [25]. However, the specific mechanism of neuronal pyroptosis in TBI has not yet been elucidated.

To better understand the mechanism of brain injury responses, we used an in vivo mouse model of TBI and in vitro cellular models of mechanical stress and inflammation. Caspase-1 knockout (KO) mice (caspase-1^−/−^), siRNA knockdown, and pharmacologic inhibition were used as tools to assess the involvement of caspase-1 in downstream inflammation following TBI and mechanical stress. We found that genetic ablation, expression reduction, and pharmacologic inhibition of caspase-1 decreased TBI- and mechanical injury-induced behavioral neurological deficits, inflammatory cytokine protein and mRNA expression levels, and neuronal apoptosis. Ultimately, our results lead us to conclude that mechanical stress caused by TBI activates caspase-1 which contributes to TBI-induced pyroptosis.

## Methods

### Animals and TBI models

In this study, we used caspase1-deficient (caspase1^−/−^; Jackson Laboratories, Bar Harbor, ME, USA) adult male mice (8–10 weeks old) and age-matched C57Bl/6 (WT) male mice. Mice were housed in the animal care facility under a 12-h light-dark cycle, with ad libitum access to food and water. All surgical procedures were carried out in accordance with protocols approved by the Institutional Animal Care and Use Committee. The animals were anesthetized with intramuscular injection of chloral hydrate (5%, 0.1 ml/10 g). Mice were fixed in a stereotaxic frame, and the scalp was shaved and cleaned with iodophor. The left lateral aspect of the skull was exposed by retracting the skin and the surrounding soft tissue. Traumatic brain injury (TBI) was induced by dropping a 200-g steel weight with a flat end from a height of 5 cm onto the left lateral skull. The free-falling weight produced a moderate contusion injury of the left parietal cortex. Sham-injured control animals underwent a similar anesthesia protocol as the TBI animals with the exception that the scalp was not exposed and no weight was dropped. About 25–30% of the mice died in the first seconds after the trauma, but there was no delayed mortality or prostration in the surviving mice. The body temperatures of the animals were monitored by a rectal probe and fixed in a range of 37 ± 0.5 °C using a heating pad. The animals were returned to their quarters after recovery from anesthesia and were allocated randomly into groups, with 18 mice in each group.

### Neurobehavioral training and evaluation

Neurological deficits were assessed using well-established, modified neurological severity scoring (mNSS), open-field and Rotarod testing at 12, 24, and 48 h after TBI. Experimenters were blinded to the TBI or sham treatments as well as the genotype of the mice. Each behavioral test was repeated twice with four different trials to validate the data. The mNSS test consists of ten different tasks that can evaluate the motor (muscle status, abnormal movement), sensory (visual, tactile, and proprioceptive), balance, and reflex functions of mice. Neurological function was graded from 0 to 18 (0 = normal function; 18 =maximal deficit). One point was scored for each abnormal behavior or for the lack of a tested reflex. Therefore, higher scores imply greater neurological injury.

The open-field test was used to determine general activity levels and to measure anxiety-like behavior. Animals were monitored under moderate lighting for 15 min in a 40-cm^2^ open field using video tracking software (ANY-Maze, Stoelting, USA). General activity was evaluated by determining the total of distance traveled. Anxiety-like behavior was assessed based on the pattern of exploration in the open field (center vs. periphery).

Fine motor coordination and learning were assessed using an accelerating Rotarod apparatus (RWD Life Science, China). On the day before the injury, mice were trained on the Rotarod for three consecutive trials at a slow rotational speed (4 rpm/min) for 1 min to adapt to the rod, followed by four additional trials with an accelerating rotational speed (from 4 to 40 rpm in 5 min) to obtain baseline latency. On each testing day, the mice were given four 300-s accelerating Rotarod trials with an inter-trial interval of 30 min. The average latency to the first fall off the rod was recorded. Passive rotation, accompanying the rotating rod without walking, were also considered as a fall.

### Primary neuronal culture and injury models

The primary neuronal culture was prepared as follow: Cortex from embryonic day 14 BALB/c mice was used for cultures. After decapitation of the mice and removal of the meninges, the cortex was digested by papayotin, and the cells were treated with DNase I (Sigma) prior to centrifugation at 1000×*g* for 3 min. Neurons were cultured on poly-l-lysine-coated dishes at a density of 1 × 10^6^ cells per 10-cm dish in minimum essential medium with 10% horse serum. The medium was changed to neuronal base medium (supplemented with 2% B27, 0.2 mM L-glutamine, and 1% penicillin-streptomycin) on the following day. The medium was changed every 3 days, and cultured cells were used for experiments on post-culture days 7–14. Subsequently, the primary neuron was collected and detected the Iba1 (microglia) and GFAP (astrocytes) expression through Western blotting (WB) and NeuN stain to assess neuronal purity.

We then performed sets of three experiments to examine the effects of mechanical and inflammatory stress on neurons in vitro: (1) neurons were incubation for 5 h with LPS and 5 mM ATP for 30 mins; (2) neurons were scratched by a 200-μl yellow gunshot; and (3) equiaxial stretch (12% strain, 1.0 Hz frequency) was applied to cultured neurons for 4 h, by a Flexcell® FX-5000™ Tension System (Flexcell, USA). In the stretch model, neurons were inoculated in 6-well plates (BioFLEX®). At the same time, we studied the role of caspase-1 in neuronal pyroptosis by caspase-1 siRNA knockdown or adding the caspase-1-specific inhibitor, Belnacasan (VX-765, 10 μM, for 24 h). After treatments, cells were used for flow cytometry, immunofluorescence, and protein or RNA extraction.

### Transient transfection

The primary neuron was respectively infected with caspase-1 shRNA (m) lentiviral particles (Santa Cruz, sc-29922-V) according to the manufacturer’s protocol (Santa Cruz Biotechnology, Inc.). Plate targets cells in a 24-well plate 48 h prior to viral infection. The cells should be approximately 50% confluent before infection. Add 0.5 ml of complete optimal medium with caspase-1 shRNA lentiviral particles (MOI = 5) (without polybrene), incubate cells for 48 h, and then change complete optimal medium without lentiviral. At 72 h after infection, the RNAi efficiency was determined by qRT-PCR (Additional file 1: Table S1 and Additional file 2: Figure S1).

### Apoptosis analysis by Annexin V-PI double staining

Neurons were stained with Annexin-V and propidium iodide (PI) using the Annexin-V-FITC kit (BD Biosciences) according to the protocol of the company. Briefly, cell culture medium was collected separately, and the treated cells were digested and washed twice with ice-cold PBS and re-suspended in the binding buffer at a concentration of 1 × 10^5^ per 100 μl. Then, the cells were supplied with 5 μl of Annexin V-FITC and 5 μl of PI and incubated in the dark at RT for 10 min. Next, 400 μl of binding buffer was added into each sample for flow cytometry analysis (BD Accuri. C6 Plus, BD Biosciences).

### LDH release detection

Mice were sacrificed under deep anesthesia for blood collection (centrifugation at 3000*g*, 10 min), and the blood serum was measured for lactate dehydrogenase (LDH) release. For the primary neuron, supernatant from serum-free media was filtered using 0.2-μm syringe filters to use for LDH release detection. The detection was using a commercially available kit (Solarbio, Beijing). One hundred microliters of the blood serum or supernatant was transferred to 96-well plates, and then the reaction mixture was added and incubated in the dark for 30 min at RT. LDH concentration was quantified by measuring the absorbance at 490 nm.

### Enzyme-linked immunosorbent assay

Total protein concentrations were measured using a BCA Protein Assay Kit (Thermo Fisher Scientific, Carlsbad, CA, USA). The levels of TGFβ, IFN-γ, IL-6, IL-10, IL-1β, and IL-18 were measured using ELISA kits (Anoric-Bio, Tianjin, China) according to the manufacturer’s instructions.

### Caspase-1 activity detection

The caspase-1 activity was detected with Caspase-1 Activity Assay Kit (Solarbio, Beijing). The specific steps were conducted according to the manufacturer’s instructions: First, 2–10 × 10^6^ cells were lysed in 50–100 μl lysis buffer on ice for 10 min. For tissue samples, 3–10 mg tissues were added to 100 μl lysis and were homogenated with a tissue homogenizer and centrifuged. The supernatant was retained. Protein concentrations were detected using the Bradford method, ensuring that the protein concentration was 1–3 μg/μl. A standard curve was prepared using the *p*NA standard. Then, the optical density of specimen was read on a microplate reader (Molecular Device) at 405 nm. The percentage of caspase-1 activity changes was calculated by the radio of OD405 of the experimental wells to that of the normal wells.

### TUNEL staining

The cerebral cortex was harvested, and apoptosis was determined by terminal deoxynucleotidyl transferase-mediated dUTP-biotin nick end labeling (TUNEL) staining, using a TUNEL Apoptosis Assay Kit (R&D, Switzerland). TUNEL staining was performed with fluorescein-dUTP for apoptotic cell nuclei and 4′,6-diamidino-2-phenylindole (DAPI) to stain all cell nuclei. apoptosis index (AI), the number of TUNEL-positive cells divided by the total cells per field, was examined. Each AI was assessed in 20 randomly selected fields.

### Immunohistochemical analysis

Mice were euthanized by injection of 5% chloralic hydras in accordance with our approved animal protocol, and the brain tissue was removed. The brain tissues were immediately perfused with 4% paraformaldehyde in PBS after removal and embedded in paraffin for sectioning. Following rehydration of the paraffin section and two washes in PBS, endogenous peroxidase activity was blocked using 3% H_2_O_2_ for 10 min. Antigen crosslinking was conducted in an autoclave for 3 min, and then the samples were washed twice in PBS. All preparations were then treated with goat serum blocking reagent (Abcam, ab7481) for 45 min. Excess reagent was removed with a quick rinse with PBS. Sections were incubated with primary antibodies overnight. Following two washes in PBS, the secondary antibody (Abgent, ASS3403) were added to all slides for 30 min. Excess reagent was removed, and the slides were washed in PBS and incubated with DAB (Solarbio, DA1010) for 5–10 min until the desired color appeared. All preparations were counterstained with hematoxylin (Solarbio, G1120) for 30 s. Protein expression was determined in five adjacent sections per sample.

### Western blot analysis

Cells were lysed in lysis buffer containing Proteinase Inhibitor Cocktail (Roche Applied Sciences) and Halt Phosphatase Inhibitor Cocktail (Thermo Scientific). Protein concentrations were quantified by using a BCA protein kit (Thermo Scientific). Samples (50 μg protein per lane) were loaded on 12% SDS-PAGE gels. After electrophoresis, the proteins were transferred to nitrocellulose membranes. Then, the membranes were blocked with 5% BSA and incubated at 4 °C overnight with the appropriate primary antibodies: caspase-1 (Abcam, 1:1000), caspase1 (p10) (AdipoGen, 1:1000), caspase-1 (p20) (AdipoGen, 1:1000), caspase-11 (Abcam, 1:1000), GSDMD (Santa Cruz, 1:100), Iba1 (Abcam, 1:1000), and GFAP (Abcam, 1:1000). Immunoreactivity was detected by incubation with horseradish peroxidase-conjugated secondary antibodies (Abgent, 1: 20,000) followed by chemiluminescent substrate development (Thermo Scientific, USA). Optical densities of the bands were calculated using a MiVnt image analysis system (Bio-Rad, CA, USA). All samples were run in parallel with four replicates.

For supernatant detection, supernatant from neurons in serum-free media was filtered using 0.2-μm syringe filters and concentrated using Centricon Plus-20 centrifugal filter devices (Millipore). Concentrated supernatant (50 μg protein per lane) was separated by 15% SDS-PAGE and transferred to nitrocellulose membranes, and processing was assessed by Western blotting using anti-caspase-1 (Abcam, 1:1000), anti-caspase1 (p10) (AdipoGen, 1:1000), and caspase-1 (p20) (AdipoGen, 1:1000). Uncropped blots for the pro-form and cleaved fragments of caspase-1 in figures were supplemented in Additional file 3: Figure S2.

To extract protein from the cortical brain tissues, the frozen brain samples were homogenized in ice-cold buffer containing 20 mM HEPES (pH 7.0), 150 mM NaCl, 1 mM EDTA, 1% Triton X-100, Proteinase Inhibitor Cocktail (Roche Applied Sciences), and Halt Phosphatase Inhibitor Cocktail (Thermo Scientific) for 30 s. The samples were sonicated through the Cell Ultrasound Cruizer (Thermo Scientific) for 10 s and centrifuged at 15,000 rpm for 10 min, and total proteins were recovered in the supernatants.

### Immunofluorescence assays

Primary neurons were subjected to immunofluorescence by the protocol described below. Cells were fixed by 4% PFA for 20 min at RT followed by PBS wash for three times (5 min each). The cells were then incubated by the blocking buffer (0.2% TritonX-100 and 5% BSA dissolved in PBS) for 1 h. After PBS wash for three times, the cells were then incubated with the primary antibodies of NeuN (Abcam, 1:300,), dissolved in the blocking buffer at 4 °C for 24 h. PBS wash was carried out followed by the treatments with the Alexa Fluor 488/568-conjugated secondary antibody (Abgent, 1:1000) dissolved in the blocking buffer for 2 h at RT. After incubation with secondary antibodies, cells were washed and counterstained with DAPI solution (1 μg/ml in the blocking buffer) for 10 min at RT. Cells were analyzed by fluorescence microscope (Leica, IX71).

### Quantitative real-time PCR

Total RNA was isolated from the cortical brain tissue or primary cultured neurons, lysed in Trizol reagent (Invitrogen) to recover the total RNA according to the manufacturer’s protocol (Trizol™ Reagent, Invitrogen). The total RNA was reversely transcripted to cDNA using a HiFi-MMLV cDNA First Strand Synthesis Kit (CW Bio, China). Quantitative real-time PCR was performed using GoTaq qPCR Master Mix (Promega) on the CFX96TM Real-Time System (Bio-Rad). GAPDH was amplified in parallel as an internal control. Primer sequences as follow, TLR4 F: 5′- TCACAACTCGCCCAAGGAGGAA -3′, R: 5′- AAGAGACCACGGCAGAAGCTAG -3′; MyD88 F: 5′- CCACCTGTAAAGGCTTCTCG -3′, R: 5′- CTAGAGCTGCTGGCCTTGTT -3′; NLRP3 F: 5′- GCTAAGAAGGACCAGCCAGAGT- 3′, R: 5′- GAACCTGCTTCTCACATGTCGT -3′; GAPDH: F: 5′- AACTTTGGCATTGTGGAAGG -3′ R: 5′- GGATGCAGGGATGATGTTCT -3′.

### Statistical analysis

All the data were presented as the mean ± SEM and were analyzed using SPSS statistical software (version 22.0, IBM). Non-parametric data from the mNSS test were analyzed using the Kruskal-Wallis *H* analysis followed by a Mann-Whitney *U* test. One-way analysis of variance (ANOVA) with repeated measures was used to analyze Rotarod data. The remaining biochemical data were analyzed using a two-way ANOVA. Each experiment was repeated three times, and significance was determined with two-tailed Student’s *t* test or one-way ANOVA. Error bars represent ± SEM. *p* value lower than 0.05 was considered as significant.

## Results

### TBI-induced neurological deficits

**Fig. 1:**
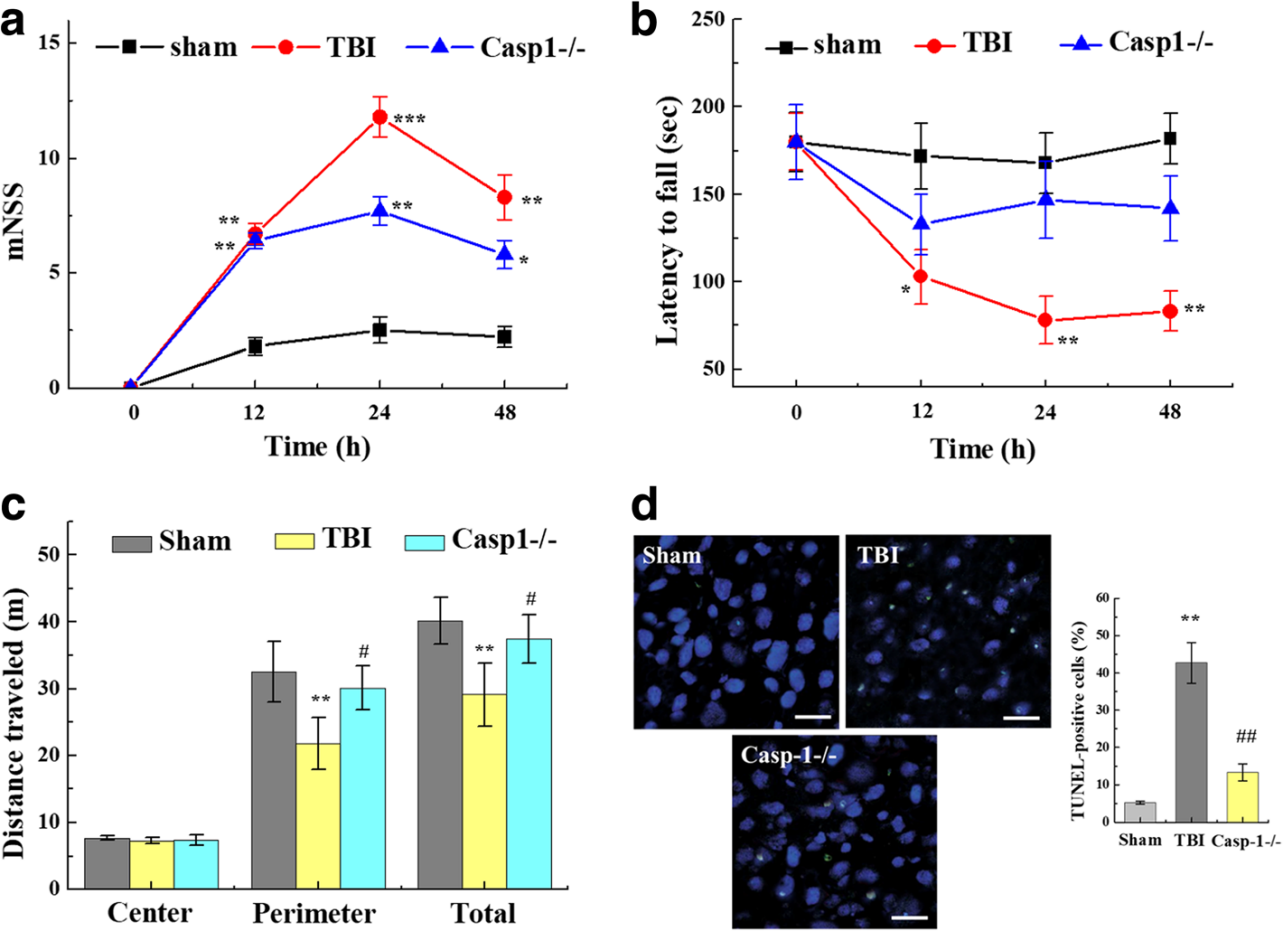
Neurological deficits after TBI. Neurological effects of TBI were assessed by **a** mNSS, **b** Rotarod, and **c** open-field behavioral task tests prior to and 12, 24, and 48 h post-TBI. **a** mNSS scores were significantly higher in the TBI groups relative to those in the sham groups. The mNSS scores were maximal at 24 h and gradually decreased with time in the TBI group. No significant difference was observed between the caspase-1^−/−^ and WT-TBI group at 12 h. However, the caspase-1^−/−^ group showed significantly lower mNSS scores relative to the WT-TBI group at 24 and 48 h post-TBI. **b** Rotarod scores were minimal at 24 h and gradually increased with time in WT-TBI group. Relative to WT-TBI mice, TBI-induced impaired Rotarod performance was attenuated in the caspase-1^−/−^ mice at 24 and 48 h after TBI. **c** In the open-field task, all mice traveled the same distance in the center zone compared to the perimeter zone. WT-TBI mice spent less time in the perimeter zone of the open-field chamber compared to caspase-1^−/−^-TBI mice. Total travel distance was significantly different between WT-TBI and caspase-1^−/−^-TBI mice. **d** TUNEL staining was used to detect cortical damage in our TBI model. Apoptosis index, the number of TUNEL-positive cells divided by the total cells per field, was examined. Each AI was assessed in 20 randomly selected fields. Data are presented as the mean ± SEM. **p < 0.01 and ***p < 0.001 versus sham group, #*p* < 0.05 and ##*p* < 0.01, compare to TBI group (*n* = 11) (mNSS, Rotarod, and open-field)

To assess neurological deficits post-TBI, we conducted a time course of behavioral testing before TBI and 12, 24, and 48 h following TBI. Prior to surgery, there was no difference in mNSS and Rotarod scores between the groups of mice. In the mNSS test (Fig. 1a), mice in the sham group showed no significant differences at 12, 24, and 48 h; however, TBI caused significantly higher mNSS relative to the sham group (*p* < 0.001). The mNSS scores were maximal at 24 h and gradually decreased, albeit insignificantly, with time in TBI group. The scores remained significantly different between the TBI and sham groups at 48 h post-injury (*p* < 0.05). Caspase-1^−/−^-TBI mice exhibited lower mNSS compared to the wide-type (WT) mice subjected to TBI (WT-TBI) (*p* < 0.01). To examine spontaneous locomotor activity in response to a novel environment, all mice were tested in the open-field behavioral task (Fig. 1c). WT-TBI mice spent a shorter time in the perimeter zone and traveled less total distance than sham group (*p* < 0.01). A modest yet statistically significant difference in perimeter zone and total travel distance was revealed between WT-TBI and caspase-1^−/−^-TBI mice (*p* < 0.05). In the Rotarod test (Fig. 1b), the sham groups exhibited the best performance relative to the TBI groups (*p* < 0.001). The scores were minimal at 24 h and following gradually increased with time in WT-TBI group. Caspase-1^−/−^-TBI mice had higher scores relative to the WT-TBI group (*p* < 0.05). The brains of the mice were assessed postmortem. TUNEL staining was used to detect cortical damage. The green fluorescence was dramatically enhanced in WT-TBI group compared to the sham group (*p* < 0.001). Apoptosis was significantly reduced in the brains of caspase-1^−/−^-TBI mice relative to the WT-TBI group (*p* < 0.05; Fig. 1d).

### Inflammatory cytokine levels in the cortex following TBI

**Fig. 2:**
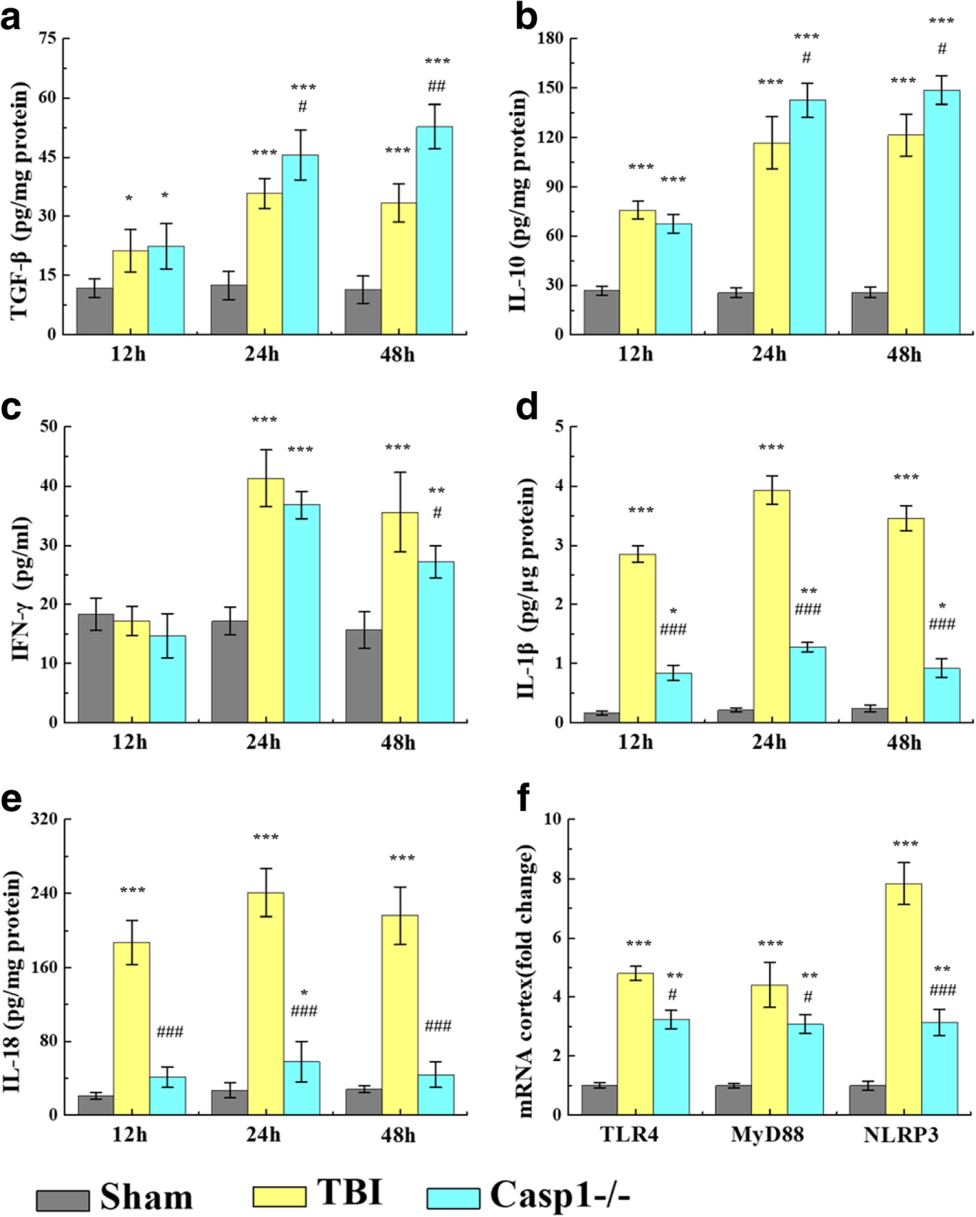
Inflammatory cytokine expression in the cortex following TBI. a–e Relative to the sham group, concentrations of the anti-inflammatory cytokines **a** TGF-β1 and **b** IL-10 and pro-inflammatory cytokines **c** IFN-γ, **d** IL-1β, and **e** IL-18 were significantly increased in the region of the contusion at 12, 24, and 48 h after TBI, whereas IFN-γ level barely had changed at 12 h after TBI. Caspase-1^−/−^-TBI mice had a less dramatic increase in anti-inflammatory cytokines and pro-inflammatory cytokines than the WT-TBI mice. **f** mRNA expression of inflammatory cytokines, *TLR4*, *MyD88*, and *NLRP3*, at 24 h post-TBI. TBI induced a marked increase in *TLR4*, *MyD88*, and *NLRP3* mRNA expression in the injured WT-TBI mice brains relative to the sham group, but caspase-1^−/−^ only exhibited mild increases. Data are presented as the mean ± SEM. **p < 0.01 and ***p < 0.001, compared to sham group, #*p* < 0.05 and ###*p* < 0.001 versus TBI, *n* = 6/group

To compare the protein levels of inflammatory-associated mediators in the cortex, an array of inflammatory cytokines were measured using ELISA. All detected cytokine levels were the lowest in the sham groups compared to the other groups (Fig. 2). Protein levels of the anti-inflammatory cytokines TGF-β at 12 h (*p* < 0.01), 24 h (*p* < 0.001), and 48 h (*p* < 0.001) and levels of IL-10 at 12 h (*p* < 0.001), 24 h (*p* < 0.001), and 48 h (*p* < 0.001) post-TBI increased in the WT-TBI group compared to the sham group (Fig. 2a, b). Furthermore, levels of the pro-inflammatory cytokines IFN-γ, IL-1β, and IL-18 were all augmented significantly at 24 h (*p* < 0.001) and 48 h (*p* < 0.01) in the TBI group relative to the sham group (Fig. 2c–e). Meanwhile, the levels of these proteins, IFN-γ, IL-1β, and IL-18, peaked at 24 h and declined significantly by 48 h (*p* < 0.05, relative to 24 h). Post-TBI at 24 h, protein levels of TGFβ and IL-10 significantly increased in injured caspase-1^−/−^ mice as compared to WT-TBI (*p* < 0.05). IFN-γ in caspase-1^−/−^-TBI mice reduced as compared to WT-TBI group (*p* < 0.05) at 48 h. There was no significant difference in protein levels of IL-1β and IL-18 in caspase-1^−/−^-TBI mice compared to sham. Gene expression related to the inflammation in the cortex region of the brain tissues was analyzed using qRT-PCR. Consistent with the ELISA results, TBI induced dramatic increases in mRNA expression of inflammatory cytokines compared to the sham groups at 24 h post-injury. The mRNA expression levels of *TLR4*, *MyD88*, and *NLRP3* all ascended at 24 h post-TBI (*p* < 0.001) (Fig. 2f). However, *NLRP3* mRNA was dramatically reduced in caspase-1^−/−^-TBI mice as compared to WT-TBI group (*p* < 0.001).

### TBI-induced pyroptosis in mice model

**Fig. 3:**
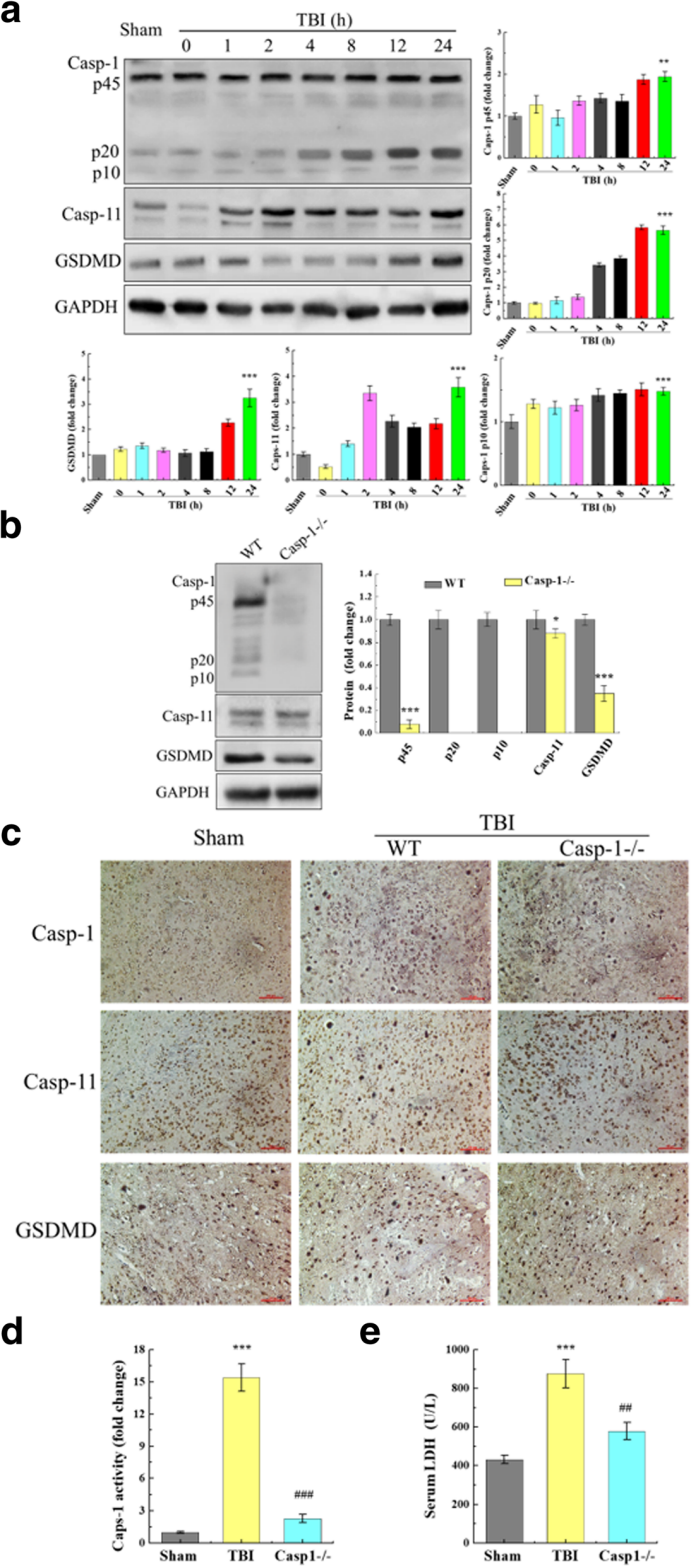
Pyroptosis in the murine model of TBI. **a** Expression of pyroptosis-related proteins in response to TBI was assessed by Western immunoblotting (WB). After 0, 1, 2, 4, 8, 12, and 24 h post-TBI, caspase-1, caspase-11, GSDMD, and caspase-1 protein fragments were severally elevated in WT-TBI mice compared to sham controls. All data are expressed as means ± SD, *n* = 5. Post-TBI at 24 h, caspase-1^−/−^ mice exhibited reduced TBI-induced pyroptosis. **b** In caspase-1^−/−^ mice, pyroptosis-related proteins were less prominent than the WT-TBI group, which demonstrated in WB results. All data are expressed as means ± SEM, *n* = 5. **c** Micrograph at × 20 magnification of caspase-1, caspase-11, and GSDMD immunostaining in the cortex in the vicinity of the lesion. As shown in the immunohistochemistry results, caspase-1, caspase-11, and GSDMD distributed in trauma organization and surrounding. The cortex showed a strong increase in caspase-1 and GSDMD staining in the WT-TBI compared to caspase-1^−/−^-TBI at 24 h post-TBI. However, caspase-11 was no obvious difference between caspase-1^−/−^-TBI and WT-TBI. One representative experiment of four was shown. **d** Caspase-1 activity was stimulated after TBI. In contrast, TBI-induced increase in caspase-1 activity was significantly reduced in caspase-1^−/−^ mice (###*p* < 0.001, WT-TBI vs. caspase-1^−/−^-TBI). **e** Serum LDH was tested at 24 h following TBI since cerebral injury increases cellular LDH leakage (*p* < 0.001). Data are presented as the mean ± SEM. *p < 0.05 and ***p < 0.001 versus sham group, ###*p* < 0.001 versus TBI group, *n* = 6

To determine whether pyroptosis was occurring in the murine TBI model and presented time-dependence, inflammation was assessed in the cortex by WB, caspase-1 activity assay kit, and immunohistochemistry. As shown in Fig. 3a, WB assay was performed to quantify pyroptosis-related protein expression at 0, 1, 2, 4, 8, 12, and 24 h post-TBI. Caspase-1 p45 was elevated at 8 h (1.4-fold), 12 h (1.9-fold), and 24 h (2.0-fold) compared to the brain tissue from uninjured animals. The p20 and p10 fragments of caspase-1 increased gradually—24 h to 5.6-folds and 1.5-folds, respectively. Meanwhile, caspase-11 and GSDMD protein expression increased after TBI. At 24 h post-TBI, caspase-11 exhibited a 3.5-fold increase, and GSDMD expression increased 3.6-fold relative to normal brain tissue. As shown in Fig. 3b, caspase-1 (including p10, p20, and p45), caspase-11, and GSDMD expression were a significant reduction in caspase-1^−/−^-TBI mice when relative to WT-TBI mice at 24 h. Immunohistochemistry was used to detect caspase-1, caspase-11, and GSDMD protein distribution in the trauma region and the surrounding brain tissue at 24 h post-TBI. As shown in Fig. 3c, caspase-1 and caspase-11 were mainly expressed in the injured area in WT-TBI mice, while they were less expressed in caspase-1^−/−^-TBI mice. They had a high degree of co-localization and gradually diffused outward radially to the surrounding tissue, and the expression gradually decreased in a radial distribution from the injury to the surrounding area. Consistent with the WB results, caspase-1 activity significantly changed after TBI. Following TBI, caspase-1 activity was significantly greater in cortical samples from TBI animals than non-injured animals (Fig. 3d). For instance, at 24 h post-injury, caspase-1 had 15.4-fold greater activity in WT-TBI mice as compared to sham controls. The caspase-1 activity was less affected in the caspase-1^−/−^-TBI mice. At 24 h post-injury, the caspase-deficient mice exhibited only a 2.3-fold increase in the activity relative to the normal brain tissue (Fig. 3d). LDH is found in almost all body tissues. It plays an important role in cellular respiration, the process by which glucose from food is converted into usable energy for cells. Although LDH is abundant in tissue cells, blood levels are normally low. While the tissues are damaged by injury, they release more LDH into the bloodstream. Interestingly, serum LDH was tested at 24 h following TBI, since cerebral injury increases cellular LDH leakage. Serum LDH concentration increased approximately 2.0-fold in WT-TBI mice relative to the sham group (*p* < 0.001) and was near normal levels in caspase-1^−/−^-TBI mice relative to the WT-TBI mice (*p* < 0.01) (Fig. 3e).

### Neuron injury-induced apoptosis

**Fig. 4:**
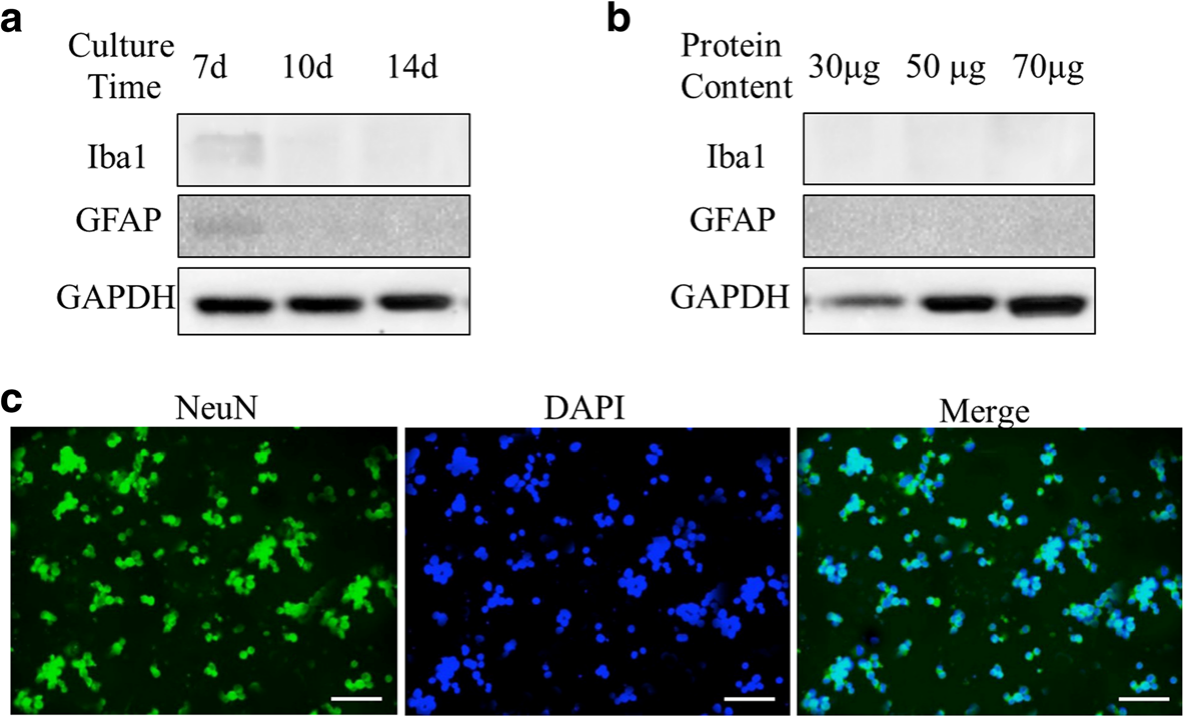
The purity of in vitro primary neuron culture. **a** Primary neuron was collected at 7, 10, and 14 days, respectively; 50 μg protein per lane was used to detect Iba1 and GFAP expression. At 7 days, Iba1 and GFAP were expressed, they were disappeared following culture at 10 and 14 days. **b** 30, 50, and 70 μg protein per lane were used to detect Iba1 and GFAP expression at 10 days. As before, there was no obvious protein bands of Iba1 and GFAP. **c** Primary neurons were stained by the neuronal marker NeuN at 10 days. To confirm the purity of in vitro primary neuron culture, cells were stained by NeuN, and DAPI was used to counterstain the cellular nucleus. Almost all cells were stained by NeuN. One representative experiment of four was shown

Primary neuron culture was potential contamination with another type of cells, such as glial cells. The primary neuron was collected and detected the Iba1 (microglia) and GFAP (astrocytes) expression through WB to assess neuronal purity. At 7 days, Iba1 and GFAP were expressed, and they were disappeared following culture at 10 and 14 days (Fig. 4a). At 10 days, there were no obvious protein bands of Iba1 and GFAP, when 30, 50, and 70 μg protein were loaded respectively (Fig. 4b). Furthermore, we have also stained the primary culture cells by markers for neurons (NeuN) and confirmed that more than 90% of cells were neurons (Fig. 4c).

**Fig. 5:**
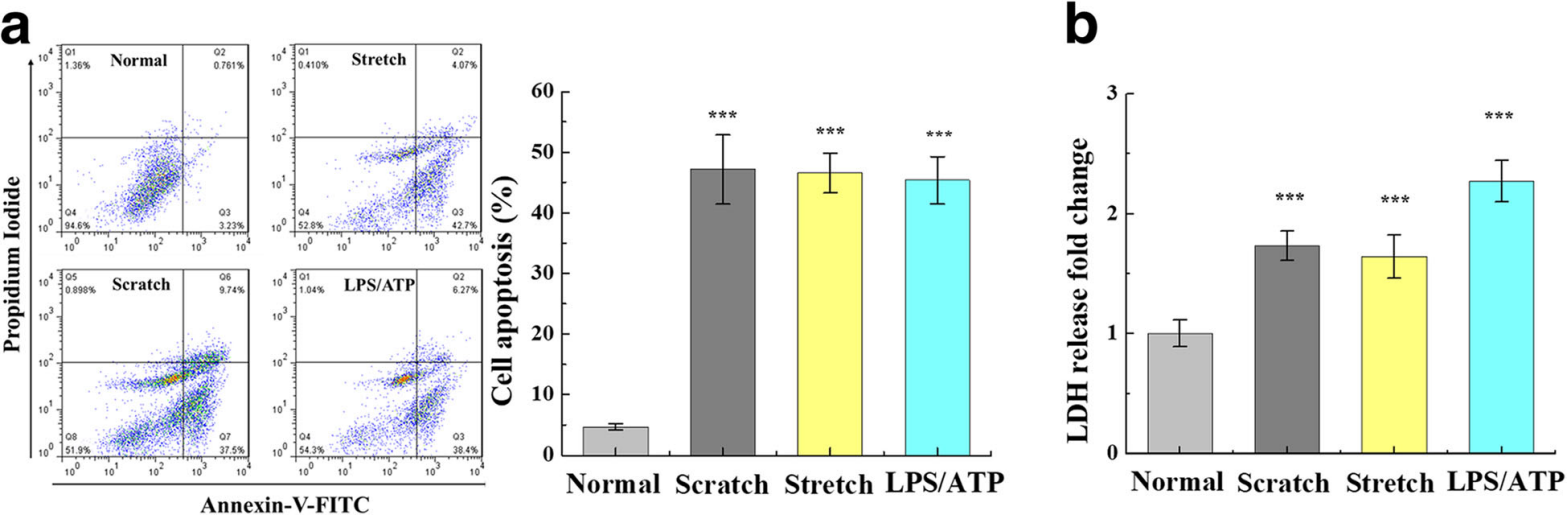
Neuron injury-induced apoptosis in cultured primary neuron. **a** Flow cytometer results for apoptosis following scratch-, stretch-, and LPS/ATP-induced neuron damage. Scratch, stretch, and LPS/ATP stimulation induced significant apoptosis in cultured primary neuron (***p < 0.001, relative to the normal group). **b** Culture supernatant was collected to detect LDH release by scratch, stretch, and LPS/ATP stimulation. Supernatant LDH concentration increased by scratch, stretch, and LPS/ATP stimulation (*p* < 0.001). All data are expressed as means ± SEM; one representative experiment of five was shown

In order to continue to explore the correlation between neuron damage and pyroptosis, we stimulated cultured primary neuron mechanically with scratch and stretch or chemically with LPS/ATP treatment. Apoptosis was detected by an Annexin-V-FITC kit. To validate these methods of mechanical and chemical injury, we assessed the apoptosis rate over time. Scratch, stretch, and LPS/ATP all significantly induced apoptosis of primary neuron (Fig. 5a). Apoptosis scores were 47.14 ± 5.51 for scratch stimulation, 46.56 ± 3.54 for equiaxial stretch, and 45.37 ± 3.87 for LPS/ATP stimulation. The culture supernatant was collected to detect LDH release by scratch, stretch, and LPS/ATP stimulation. Supernatant LDH concentration increased approximately 1.7-fold, 1.6-fold, and 2.2-fold by scratch, stretch, and LPS/ATP stimulation, respectively, relative to normal group (*p* < 0.001) (Fig. 5b).

### Neuron injury potentially induced inflammation and pyroptosis

**Fig. 6:**
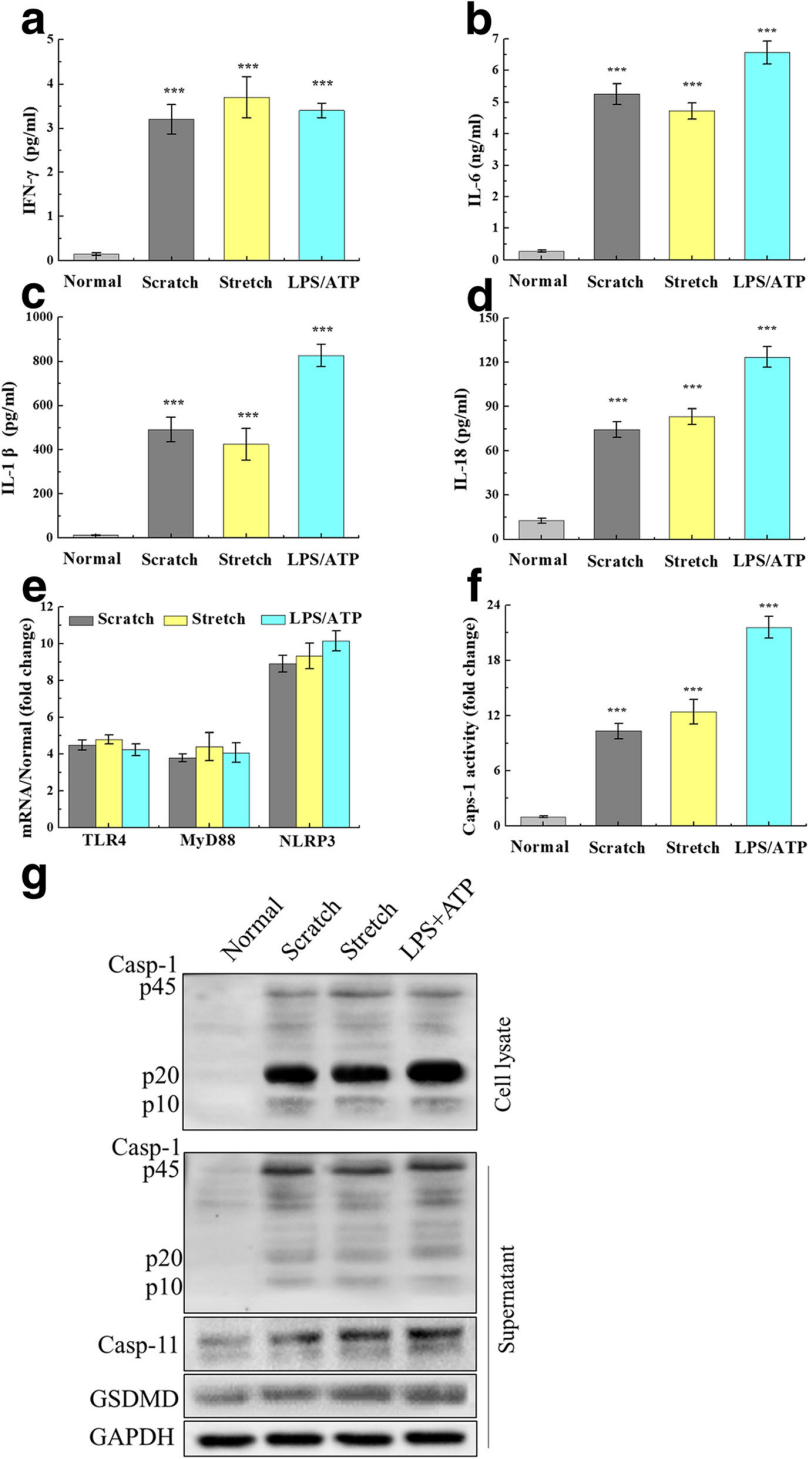
Neuron injury-induced inflammation and pyroptosis. Relative to the control group (cultured primary neuron without mechanical or chemical treatment), scratch, stretch, and LPS/ATP stimulation induced significant increases in pro-inflammatory cytokines: **a** IFN-γ, **b** IL-6, **c** IL-1β, and **d** IL-18. **e** mRNA expression of *TLR4*, *MyD88*, and *NLRP3* after scratch, stretch, and LPS/ATP treatments. Expression of these transcripts increased by 4.5-fold, 4.0-fold, and 9.0-fold, respectively, compared to control neurons. **f** Caspase-1 activity was enhanced at 10.3-fold, 12.4-fold, and 21.6-fold following scratch, stretch, and LPS/ATP stimulation, compared to the control group. **g** Cell lysate and culture supernatant were collected; pyroptosis-related protein expression was detected by WB after scratch, stretch, and LPS/ATP stimulation in cultured primary neuron. Caspase-1 and its fragments (p10 and p20) leaked to the culture supernatant. The histogram was used to analyze protein expression in the cell lysate. Data are presented as the mean ± SEM. ***p < 0.001 versus normal group; one representative experiment of five was shown

ELISA and qRT-PCR were used to determine the expression of inflammatory mediators following scratch, stretch, or LPS/ATP stimulation of primary neuron. Scratch, stretch, and LPS/ATP stimulation significantly increased secretion of IFN-γ, IL-6, IL-1β, and IL-18 compared to untreated neurons (*p* < 0.001; Fig. 6a–d). IL-1β was secreted at 12.76 ± 1.37 pg/ml from untreated neurons and at levels of 491.54 ± 56.33 pg/ml (scratch), 424.86 ± 71.36 pg/ml (equiaxial stretch), and 825.63 ± 51.16 pg/ml (LPS/ATP) in the stimulation groups. IL-18 was secreted at 12.14 ± 1.81 pg/ml from untreated neurons, whereas stimulation caused increases in levels to 74.52 ± 5.33 pg/ml (scratch), 83.42 ± 5.36 pg/ml (equiaxial stretch), and 123.67 ± 7.16 pg/ml (LPS/ATP). *TLR4*, *MyD88*, and *NLRP3* mRNA were analyzed using qRT-PCR (Fig. 6e). Neuron injury from scratch, stretch, and LPS/ATP stimulation induced dramatic increases in the expression: *TLR4* mRNA increased 4.5-fold, *MyD88* mRNA 4.0-fold, and *NLRP3* mRNA 9.0-fold relative to untreated neurons (Fig. 6e). Expression of *NLRP3* was remarkably higher than *TLR4* and *MyD88* (*p* < 0.001). Significant changes in caspase-1 activity were detected using the Caspase-1 Activity Assay Kit, and pyroptosis-related protein expression was also detected using a WB assay. Primary neuron was lysed to detect caspase-1 activity after scratch, stretch, or LPS/ATP stimulation. Caspase-1 activity was increased, respectively, 10.3-, 12.4-, and 21.6-fold relative to normal culture (*p* < 0.001) (Fig. 6f). In separate samples, WB assays were performed to quantify pyroptosis-related protein expression. As shown in Fig. 6g, mechanical scratch, equiaxial stretch, and LPS/ATP stimulation enhanced the expression of caspase-1, caspase-11, and GSDMD. Moreover, there leaked a large number of caspase-1 p45 and its fragments (p10 and p20) in the culture supernatant.

### Caspase-1 effected neuron injury

**Fig. 7:**
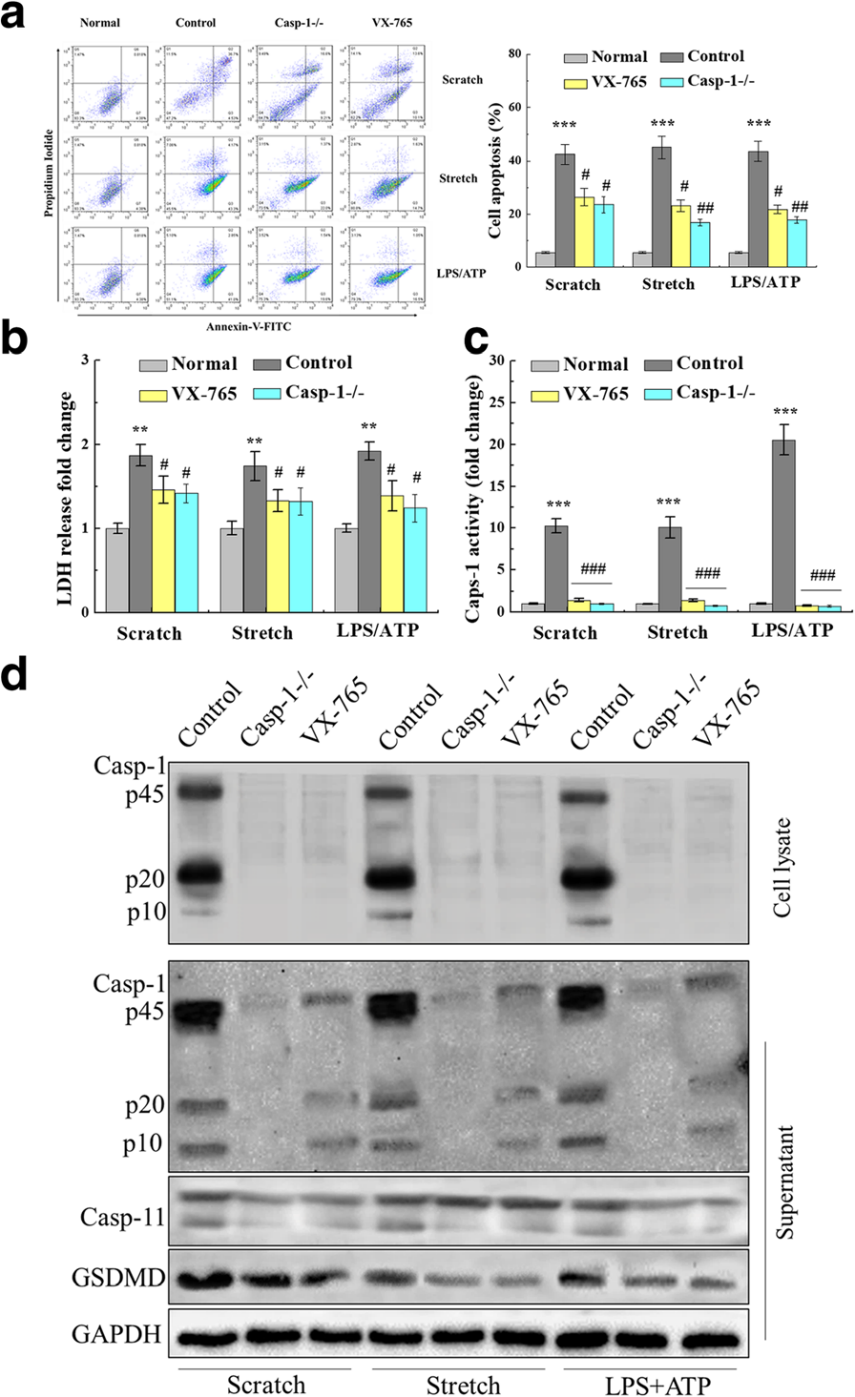
Caspase-1 deficiency attenuated neuron injury-induced apoptosis and pyroptosis. **a** Mechanical scratch, equiaxial stretch, and LPS/ATP stimulation induced apoptosis and caspase-1 knockdown with siRNA and pharmacologic inhibition with VX-765 reduced cellular apoptosis following scratch, stretch, and LPS/ATP. **b** Supernatant LDH concentration was detected when caspase-1 was a deficiency (siRNA and VX-765). **c** Caspase-1 activity was enhanced with scratch, stretch, and LPS/ATP stimulation of cultured primary neuron. Caspase-1 deficiency reduced the caspase-1 activity enhancement. **d** Caspase-1 deficiency decreased pyroptosis-related protein expression and reduced caspase-1 and its fragments (p10 and p20) leakage. The histogram was used to analyze protein expression in the cell lysate. Data are presented as the mean ± SEM. **p < 0.01 and ***p < 0.001 versus normal group, #*p* < 0.05, ##*p* < 0.01, and ###*p* < 0.001 versus control group, one representative experiment of five was shown

In order to determine whether caspase-1 resides in upstream cellular signaling pathways that lead to pyroptosis after mechanical and chemical stress and injury, we utilized caspase-1 siRNA to knockdown expression of caspase-1 as well as a pharmacological inhibitor, VX-765. Caspase-1 siRNA and inhibition with VX-765 reduced neuron apoptosis following mechanical scratch, equiaxial stretch, and LPS/ATP stimulation (Fig. 7a). After VX-765 treatment, apoptosis rates were 26.36 ± 3.16 cells/h for scratch, 23.13 ± 2.13 cells/h for equiaxial stretch, and 21.79 ± 1.51 cells/h for LPS/ATP. Similarly, with siRNA knockdown of caspase-1, apoptosis scores were 23.54 ± 3.11 cells/h for scratch, 16.82 ± 1.28 cells/h with equiaxial stretch, and 17.72 ± 1.11 cells/h with LPS/ATP. In comparison, the apoptosis scores for neurons with fully functional caspase-1 were significantly greater—41.36 ± 6.44 cells/h for scratch, 47.38 ± 4.61 cells/h for equiaxial stretch, and 44.62 ± 4.81 cells/h for LPS/ATP. The culture supernatant was collected to detect LDH release. Supernatant LDH concentration observably increased after the scratch, stretch, or LPS/ATP stimulation, relative to normal culture (*p* < 0.01), whereas the leaked LDH significantly reduced in caspase-1 siRNA and VX-765 groups, relative to control group (*p* < 0.05) (Fig. 7b). Enhanced caspase-1 activity by neuronal injury was inhibited by caspase-1 siRNA and VX-765 inhibition (Fig. 7c). Expression of caspase-1, caspase-11, and GSDMD decreased in caspase-1 siRNA and VX-765 groups, and caspase-1 p45 and its fragments (p10 and p20) significantly reduced in the culture supernatant, simultaneously (Fig. 7d). In order to demonstrate the reduction in the pro-form of caspase-1 levels by VX-765 was not an artifact due to direct VX-765 neurotoxicity, VX-765 groups, and a vehicle control (DMSO as the menstruum) for VX-765 treated with neuron. CCK8 results demonstrated that there was no neurotoxicity by VX-765 at 10 μM (Additional file 4: Figure S3). Further, ELISA results showed decreased secretion of neuroinflammatory mediators, such as IL-1β and IL-18, when the caspase-1 was inhibited (Table 1).

**Table 1:** Secretions of inflammatory cytokines were measured by ELISA

Cytokine	Scratch			Stretch			LPS/ATP		
	Control	VX-765	Casp-1−/−	Control	VX-765	Casp-1−/−	Control	VX-765	Casp-1−/−
IFN-γ (U/ml)	3.56 ± 0.46	3.03 ± 0.23*	3.16 ± 0.18*	3.72 ± 0.28	2.87 ± 0.14**	2.73 ± 0.26**	3.44 ± 0.16	2.31 ± 0.22**	2.27 ± 0.17**
IL-6 (ng/ml)	5.37 ± 0.41	3.01 ± 0.27***	3.14 ± 0.35***	4.67 ± 0.26	2.71 ± 0.18***	2.63 ± 0.26***	6.62 ± 0.47	3.64 ± 0.26***	3.41 ± 0.37***
IL-1β (pg/ml)	483.26 ± 34.22	73.36 ± 5.16***	53.43 ± 4.66***	428.38 ± 56.71	63.17 ± 3.09***	51.27 ± 4.57***	803.52 ± 38.14	55.27 ± 3.56***	43.71 ± 4.06***
IL-18 (pg/ml)	68.47 ± 4.46	11.63 ± 0.27***	6.47 ± 0.38***	77.73 ± 5.04	9.38 ± 0.17***	7.36 ± 0.43***	122.51 ± 4.18	8.67 ± 0.57***	7.74 ± 0.39***

Data are presented as the mean ± SEM

*p < 0.05, **p < 0.01, and ***p < 0.001 versus control group, one representative experiment of five was shown

## Discussion

Results of this study demonstrate that pyroptosis modulates responses to the acute phase of TBI through caspase-1. Caspase-1 is expressed and activated in neurons and therefore is likely to be activated via the mechanical damage. During the acute post-traumatic period (0–48 h), neurological deficits, inflammatory cytokine, and pyroptosis are greater in the brain-injured mice. To date, there are scant data regarding the regulating of pyroptosis during the acute phase of TBI. Therefore, we performed a time-effect study for damage on post-TBI sensory-motor deficits and motor dysfunctions, which has significantly higher mNSS score and minimum Rotarod test score at 24 h. We found that acute injury significantly reduced neuron functional deficits as early as 24 h post-TBI. For instance, a previous study using this murine TBI model observed higher neurological deficits at 24 h than at 72 h and found that brain injury is most pronounced after 24 h [26]. Inflammatory correlation factors are also detected in the acute phase. A range of anti-inflammatory cytokines and pro-inflammatory cytokine secretion were all increased and continued high secretion from 24 to 48 h, such as TGF-β, IL-10, IFN-γ, IL-1β, and IL-18. Simultaneously, mRNA of the upstream inflammatory regulator TLR4, MyD88, and NLRP3 are also highly expressed at 24 h post-TBI. Apoptosis and immunostaining of the damaged cortex further validated our murine TBI model. There was significant apoptosis, expression of caspase-1, caspase-11, and GSDMD proteins around the contusion site, and blood LDH release at 24 h post-TBI. These data suggest that pyroptosis is likely involved in neuroinflammation with TBI. Apoptoses mediated by caspase-induced cleavage events are key features of pyroptosis [27]. Adamczak et al. verify neuronal pyroptosis which is mediated by the AIM2 inflammasome, is an important cell death mechanism during the CNS infection and injury [28]. NLRP3 inflammasome plays a key role in the secondary phase of TBI, and lack of the NLRP3 inflammasome will improve recovery following TBI [29–31]. AIM2 and NLRP3 as sensors, they are involved in activation of caspase-1 which catalyze neuroinflammation and pyroptosis. However, we directly demonstrate whether pyroptosis involves the acute phase of TBI and whether caspase-1 deficiency will reduce neuroinflammation and neuron injury in vivo and in vitro.

Pyroptosis drives by noncanonical and canonical inflammasomes [32]. In the process of pyroptosis, Toll-like receptor (TLR)- and/or interferon (IFN)-mediated priming upregulates the expression of guanylate binding proteins critical for bacterial vacuole lysis and/or pattern-associated molecular pattern (PAMP, including LPS and bacterial DNA) exposure, sensors (NLRP3, caspase-1, caspase-11), and cytokine precursor (pro-IL-1β, pro-IL-18) [33, 34]. The ability of inflammatory caspases to promote cell lysis involves the cleavage of GSDMD to promote the formation of membrane pores [35]. Membrane disruption leads to pyroptosis lytic cell death, releasing alarmins and pro-inflammatory cytokines [36, 37]. Noncanonical inflammasome also controls caspase-1 activation mediated by the NLRP3 canonical inflammasome through pannexin-1 and GSDMD cleavages in a cell-intrinsic manner involving K^+^ efflux [38–40]. Furthermore, some studies have shown that the pyroptosis is usually along with the released cytoplasmic LDH [41, 42]. All those suggest that pyroptosis involves in the acute phase of TBI, through increasing caspase-1 activity, caspase-11, GSDMD expression, and blood LDH release. While in caspase-1^−/−^-TBI mice, caspase-1 deficiency impels pyroptosis decay, less LDH release, neuroinflammation, neurological deficits, and neuronal death in the acute phase.

On the cell level, we stimulated primary neuron through scratch, stretch, or LPS/ATP, to discuss the mechanism of pyroptosis on TBI. Mechanical scratch can directly damage the neurons; inflammatory cytokines in damaged neurons may release rapidly and thus affect the activity of other normal neurons. As for the mechanical stretch, it is commonly used to study the biomechanical stimulation of cardiovascular disease and bone-related diseases [43, 44]. The neuron/axles may be damaged under equiaxial stretch (12% strain, 1.0 Hz frequency) for 4 h. In some reports, LPS usually has been used to induce inflammation and pyroptosis of macrophages [23, 36]. In this work, we tried to incur pyroptosis by LPS/ATP in the primary neuron. Scratch or stretch and LPS/ATP, all caused neuron damage, neuron apoptosis, LDH leakage, and a lot of inflammatory cytokine release, and pyroptosis-related proteins were also triggered. Caspase is a protease involved in cell apoptosis, with more than ten subtypes. Caspase-1 regulates cell apoptosis by shearing Bcl-XL and the inflammatory response of related cytokines which are mediated by the shear of the precursors of cytokines [45]. Caspase-1 is the unique caspase that produces active cytokines in the precursors of IL-1β and IL-18 [19]. Caspase-11 shears 45kd-caspase-1 precursor protein to produce p10 and p20 fragments, which form a heterogenous dimer and then form a tetrameric active enzyme [46]. In the case of caspase-1 suppression, caspase-1 activity was significantly inhibited, it weakens the injure and LDH leakage of neurons which induced by scratch, stretch, or LPS/ATP, and it decreased the expression or the release of inflammatory factors. Although previous studies had suggested that caspase-1 was the main enzyme implicated in the cleavage of pro-IL-1β and pro-IL-18 into the biologically active cytokine, caspase-1 and its fragments (p10 and p20) became less and mature of IL-1β and IL-18 reduced when caspase-1 was suppressed in the primary neuron. In the meantime, caspase-1 suppression aroused decrease of GSDMD, which formed large pores in the membrane that drive swelling and membrane rupture. Ultimately, caspase-1 deficiency reduced secretion of mature IL-1β and IL-18, which abated neuroinflammation [47].

Our results align with what is known about the role of caspase-1 in other brain injury responses. Previous researches show that caspase-1 KO mice and transgenic mice expressing a dominant-negative caspase-1 construct are partially resistant to cerebral disease and stroke. In the permanent stroke model, the proteolytic activity of caspase-1 is increased 30 min after occlusion with a second wave of activation 12 h later [48]. The increase of caspase-1 expression has been described in neurons and astrocytes after thromboembolic stroke and observed later in microglia (24 h post-stroke) [49]. Other studies have demonstrated the role of caspase-1 after stroke using transgenic mice. Caspase-1 KO mice and mice expressing dominant-negative caspase-1 exhibit a reduction of brain damage relative to wild type after experimental stroke [50]. Moreover, intracerebroventricular administration of caspase-1 inhibitors provides protective effects in experimental stroke models [51, 52]. Beneficial effects of caspase-1 inhibition are also observed in a model of oxygen and glucose deprivation in rat organotypic hippocampal slices [53]. Some studies indicate that the caspase-1 mRNA expression in the cortex was significantly increased in AD patients [54]. Altogether, these studies as well as our study show that caspase-1 is a key regulator of underlying mechanisms of brain injury and disease.

Our study experimentally demonstrates that TBI induces pyroptosis, and caspase-1 deficiency reduces neuroinflammation and neuronal damage in the acute phase. These findings may provide strategies for clinical treatment of TBI and other neuroinflammatory conditions. There is an increasing realization that neuroinflammation occurs within the whole gamut of the central nervous system pathologies [55]. Regulating inflammation to control TBI damage is possible through drug treatments [56]. NLRP3 inflammasome and interleukin-1 receptor antagonist treatments have been used to mitigate neuroinflammation after TBI [30, 57]. These treatments were able to alleviate the nerve damage and promote neurological recovery after TBI. Electro-acupuncture-induced TLR4 signaling inhibition has been used to promote hippocampal neurogenesis and neurological recovery post-trauma [58].

## Conclusion

Overall, our findings indicate that TBI induces pyroptosis, and caspase-1 is a significant player in neuroinflammation. The caspase-1 deficiency drives an anti-inflammatory response in the injured cortex, and depressed pyroptosis alleviates some of the neuroinflammation and neurological deficits involved in the acute phase of TBI. Therefore, inhibition of caspase-1 may alleviate the severity of TBI and improve the efficacy of these other blocking agents.

## Additional files

Additional file 1: Table S1. Primers of caspase-1 for RT-qPCR. (DOCX 15 kb)

Additional file 2: Figure S1. Caspase-1 mRNA expression level in the primary neuron. The primary neuron was infected with caspase-1 shRNA (m) lentiviral particles (Santa Cruz, sc-29922-V) according to the manufacturer’s protocol (Santa Cruz Biotechnology, Inc.). At 72 h after infection, The RNAi efficiency was determined by qRT-PCR. Caspase-1 mRNA was significantly reduced by RNAi. Data were represented as means ± SEM, one representative experiment of four was shown. ***p < 0.001, versus control. (DOCX 24 kb)

Additional file 3: Figure S2. Uncropped blots for the pro-form and cleaved fragments of caspase-1 in figures. (DOCX 877 kb)

Additional file 4: Figure S3. VX-765 neurotoxicity detected by CKK8 assay. In order to demonstrate the reduction in the pro-form of caspase-1 levels by VX-765 was not an artifact due to direct VX-765 neurotoxicity, a VX-765 alone and a vehicle control for VX-765 treated with neuron. The primary neuron was seeded in 96-well according to the primary neuronal cultures. At 10 days, cells were supplied with a fresh medium and treated with VX-765 (0, 1, 5, and 10 μM). After 24 h of cultivation respectively, then 10 μl of the CCK8 reaction solution (Dojindo Laboratories) was added to each well. After 4 h incubation at 37 °C in the 5% CO2 incubator, the absorbance at 450 nm was measured by Thermo MK3 (Thermo Scientific). The primary neuron was supplied with a fresh medium and treated with VX-765 at 0, 1, 5, and 10 μM (DMSO as the menstruum). After 24 h of cultivation respectively, neurotoxicity was detected by CCK8 assay. Data were represented as means ± SEM, one representative experiment of five was shown. (DOCX 43 kb)

## Abbreviations

AD: Alzheimer disease
ANOVA: Analysis of variance
Aβ: Amyloid-beta peptide
Bach: BTB and CNC homology
BCA: Bicinchoninic acid
DAB: 3,3′-Diaminobenzidine solution
DAPI: 4′,6-Diamidino-2-phenylindole
DNA: Deoxyribonucleic acid
ELISA: Enzyme-linked immunosorbent assay
Erk: Extracellular regulated protein kinases
FITC: Fluorescein isothiocyanate
GAPDH: Glyceraldehyde-3-phosphate dehydrogenase
GSDMD: Gasdermin D
HRP: Avidin-biotin horseradish peroxidase
IFN: Interferon
IL: Interleukin
KO: knockout
LPS: Lipopolysaccharide
mNSS: Modified neurological severity score
MyD: Myeloid differentiation primary-response gene
NLRP: Nucleotide-binding oligomerization domain, leucine rich repeat, and pyrin domain containing
Nrf: NF-E2-related factor
PBS: Phosphate-buffered saline
PCR: Polymerase chain reaction
PI: Propidium iodide
Rick: Receptor-interacting serine-threonine kinase
Rip: Receptor-interacting protein
RNA: Ribonucleic acid
SAH: Stable single α-helices
SD: Standard deviation
siRNA: Small interfering RNA
TBI: Traumatic brain injury
TGF: Transforming growth factor
TLR: Toll-like receptors
TNF: Tumor necrosis factor
TUNEL: Terminal deoxynucleotidyl transferase-dUTP nick end labeling
WB: Western blotting
WT: Wide-type
