# Supplementary material for: Ablation of caspase-1 protects against TBI-induced pyroptosis in vitro and in vivo
Source: J Neuroinflammation. 2018 Feb 19;15:48. doi: 10.1186/s12974-018-1083-y (PMC5817788; doi:10.1186/s12974-018-1083-y)
Supplement: Supplementary file 1 — Primers of caspase-1 for RT-qPCR. (DOCX 15 kb) [file 12974_2018_1083_MOESM1_ESM.docx]

**Table S1. Primers for RT-qPCR**

| **Primer** | **Sequence（5'-3'）** |
| --- | --- |
| Casp-1 -F | CTTGGAGACATCCTGTCAGGG |
| Casp-1 -R | AGTCACAACACCAGGCATATT |
