# Supplementary figures and images for: Ablation of caspase-1 protects against TBI-induced pyroptosis in vitro and in vivo
Source: J Neuroinflammation. 2018 Feb 19;15:48. doi: 10.1186/s12974-018-1083-y (PMC5817788; doi:10.1186/s12974-018-1083-y)

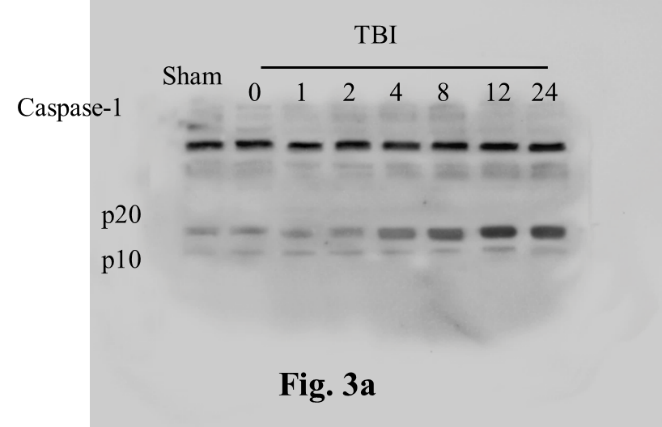


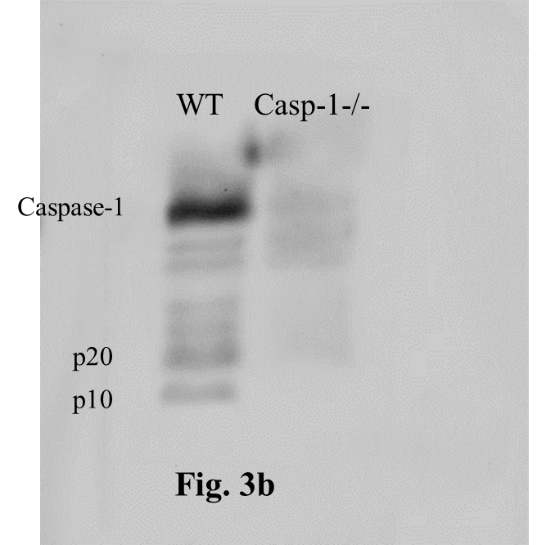


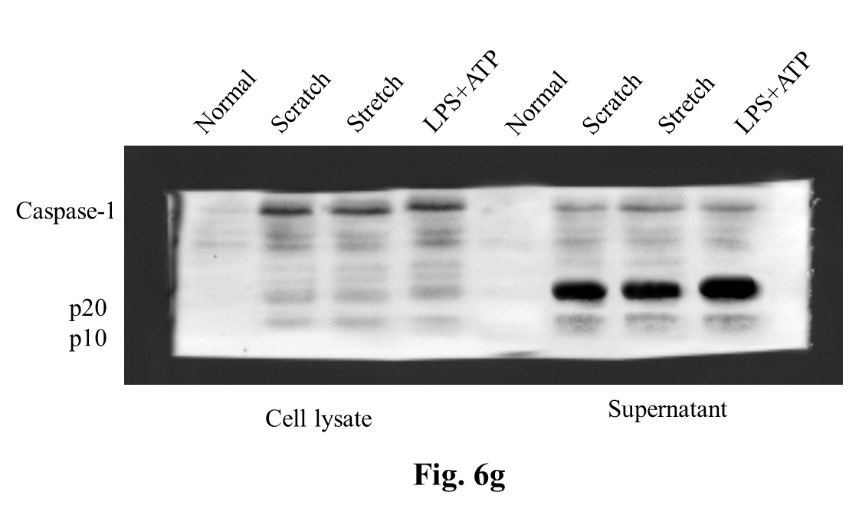


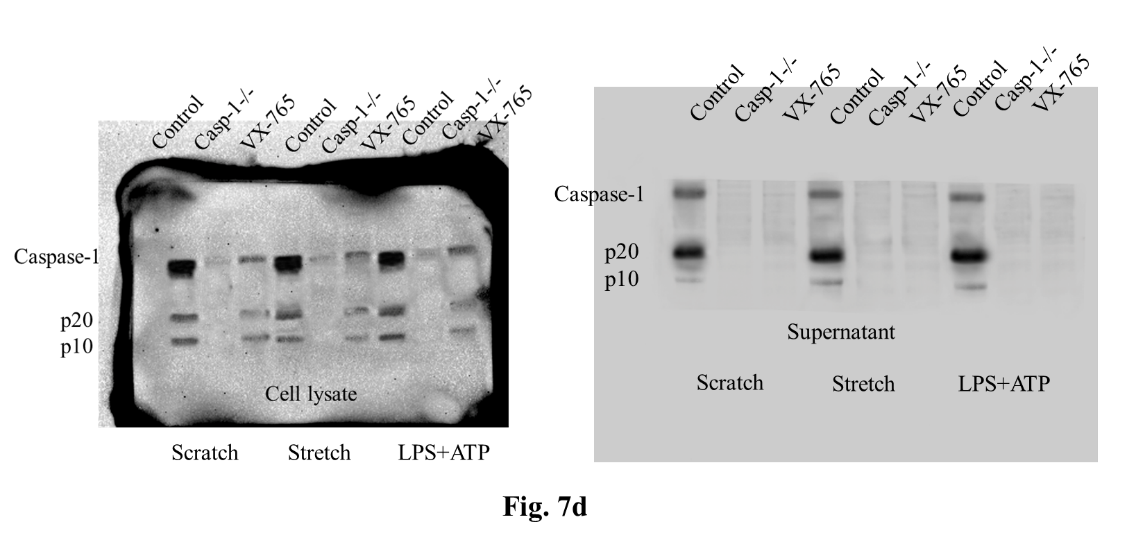

Supplement: Supplementary file 3 — Uncropped blots for the pro-form and cleaved fragments of caspase-1 in figures. (DOCX 877 kb) [file 12974_2018_1083_MOESM3_ESM.docx]

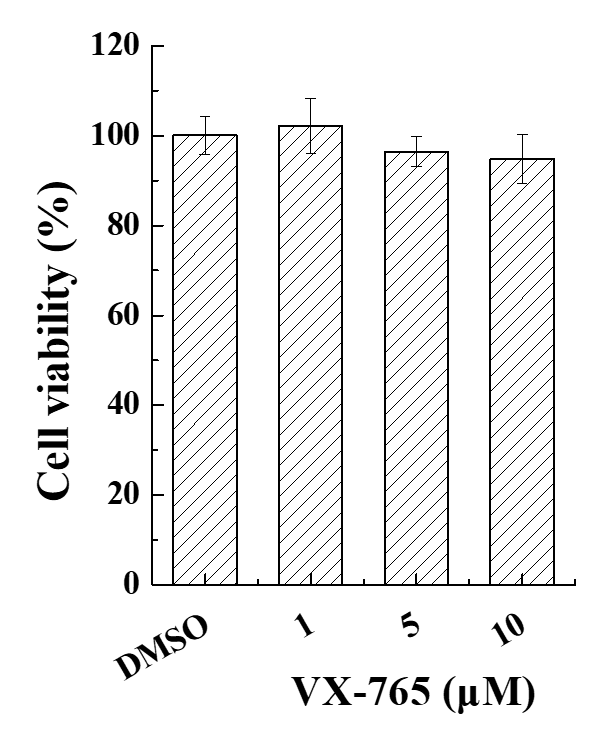

Supplement: Supplementary file 4 — VX-765 neurotoxicity detected by CKK8 assay. In order to demonstrate the reduction in the pro-form of caspase-1 levels by VX-765 was not an artifact due to direct VX-765 neurotoxicity, a VX-765 alone and a vehicle control for VX-765 treated with neuron. The primary neuron was seeded in 96-well according to the primary neuronal cultures. At 10 days, cells were supplied with a fresh medium and treated with VX-765 (0, 1, 5, and 10 μM). After 24 h of cultivation respectively, then 10 μl of the CCK8 reaction solution (Dojindo Laboratories) was added to each well. After 4 h incubation at 37 °C in the 5% CO2 incubator, the absorbance at 450 nm was measured by Thermo MK3 (Thermo Scientific). The primary neuron was supplied with a fresh medium and treated with VX-765 at 0, 1, 5, and 10 μM (DMSO as the menstruum). After 24 h of cultivation respectively, neurotoxicity was detected by CCK8 assay. Data were represented as means ± SEM, one representative experiment of five was shown. (DOCX 43 kb) [file 12974_2018_1083_MOESM4_ESM.docx]
