# Supplementary material for: Ablation of caspase-1 protects against TBI-induced pyroptosis in vitro and in vivo
Source: J Neuroinflammation. 2018 Feb 19;15:48. doi: 10.1186/s12974-018-1083-y (PMC5817788; doi:10.1186/s12974-018-1083-y)
Supplement: Supplementary file 3 [file 12974_2018_1083_MOESM2_ESM.docx]

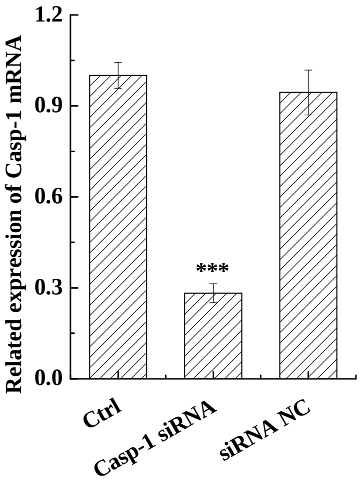
Related expression of Casp-1 mRNA
1.2
0.9
0.6
0.3
0.0
***
Ctrl
Casp-1 siRNA
siRNA NC
